# Biofouling on titanium implants: a novel formulation of poloxamer and peroxide for *in situ* removal of pellicle and multi-species oral biofilm

**DOI:** 10.1093/rb/rbae014

**Published:** 2024-02-10

**Authors:** Badra Hussain, Roger Simm, Jaime Bueno, Savvas Giannettou, Ali-Oddin Naemi, Ståle Petter Lyngstadaas, Håvard Jostein Haugen

**Affiliations:** Department of Biomaterials, Institute of Clinical Dentistry, University of Oslo, Oslo, Norway; Institute of Oral Biology, University of Oslo, Oslo, Norway; Department of Biomaterials, Institute of Clinical Dentistry, University of Oslo, Oslo, Norway; Section of the Postgraduate program in Periodontology, Faculty of Dentistry, Complutense University, Madrid (UCM), Madrid, Spain; Department of Biomaterials, Institute of Clinical Dentistry, University of Oslo, Oslo, Norway; Institute of Oral Biology, University of Oslo, Oslo, Norway; Department of Biomaterials, Institute of Clinical Dentistry, University of Oslo, Oslo, Norway; Department of Biomaterials, Institute of Clinical Dentistry, University of Oslo, Oslo, Norway

**Keywords:** chemical decontamination, cleaning of implant surfaces, complex oral biofilms, pellicle formation, peri-implantitis

## Abstract

Eradicating biofouling from implant surfaces is essential in treating peri-implant infections, as it directly addresses the microbial source for infection and inflammation around dental implants. This controlled laboratory study examines the effectiveness of the four commercially available debridement solutions ‘(EDTA (Prefgel^®^), NaOCl (Perisolv^®^), H_2_O_2_ (Sigma-Aldrich) and Chlorhexidine (GUM^®^ Paroex^®^))’ in removing the acquired pellicle, preventing pellicle re-formation and removing of a multi-species oral biofilm growing on a titanium implant surface, and compare the results with the effect of a novel formulation of a peroxide-activated ‘Poloxamer gel (Nubone^®^ Clean)’. Evaluation of pellicle removal and re-formation was conducted using scanning electron microscope (SEM), energy-dispersive X-ray spectroscopy and X-ray photoelectron spectroscopy to assess the surface morphology, elemental composition and chemical surface composition. Hydrophilicity was assessed through contact angle measurements. The multi-species biofilm model included *Streptococcus oralis*, *Fusobacterium nucleatum* and *Aggregatibacter actinomycetemcomitans*, reflecting the natural oral microbiome’s complexity. Biofilm biomass was quantified using safranin staining, biofilm viability was evaluated using confocal laser scanning microscopy, and SEM was used for morphological analyses of the biofilm. Results indicated that while no single agent completely eradicated the biofilm, the ‘Poloxamer gel’ activated with ‘H_2_O_2_’ exhibited promising results. It minimized re-contamination of the pellicle by significantly lowering the contact angle, indicating enhanced hydrophilicity. This combination also showed a notable reduction in carbon contaminants, suggesting the effective removal of organic residues from the titanium surface, in addition to effectively reducing viable bacterial counts. In conclusion, the ‘Poloxamer gel + H_2_O_2_’ combination emerged as a promising chemical decontamination strategy for peri-implant diseases. It underlines the importance of tailoring treatment methods to the unique microbial challenges in peri-implant diseases and the necessity of combining chemical decontaminating strategies with established mechanical cleaning procedures for optimal management of peri-implant diseases.

## Graphical abstract

**Figure:**
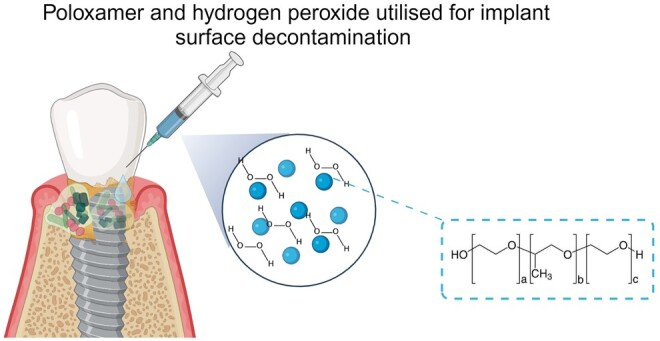

## Introduction

Dental implants have revolutionized the way missing teeth are replaced, and they have now become a widely accepted and frequently used treatment option for tooth loss [1, 2], with excellent success rates in osseointegration [3, 4]. Osseointegration is a biological process whereby a direct interface is formed between an implant and bone, without intervening soft tissue [5, 6]. One of the biggest problems associated with dental implants is peri-implantitis [7, 8]. Peri-implantitis is defined as a destructive inflammatory lesion with an accelerating pattern affecting the peri-implant bone of the implants in function [8, 9], all of which are initially osseointegrated. The progression of this disease will eventually lead to implant loss.

Biofilm is widely recognized as the primary cause of peri-implantitis [9, 10]. Biofilm formation begins with forming a pellicle layer on the surface, which serves as a foundation for bacterial attachment and subsequent biofilm maturation [11]. The adhesion of proteins to the surface facilitates bacterial attachment and biofilm development [11]. The exposure of the implant to the oral cavity places these implants in a unique position compared to orthodontic implants. The goal is to prevent biofilm formation or eradicate it once established.

The aetiology of peri-implantitis is intricately associated with multi-species biofilms that adhere to dental implants [12]. For a nuanced understanding of peri-implantitis, employing a multiple-species biofilm model that includes pivotal oral bacteria such as *Streptococcus oralis*, *Fusobacterium nucleatum* and *Aggregatibacter actinomycetemcomitans* is critical [13–15]. These species are integral to the natural biofilm development within the oral cavity and play distinct roles in the progression of peri-implantitis, making them more clinically relevant than the frequently utilized *Staphylococcus epidermidis*, which, despite the prevalence in nosocomial infections, do not typically inhabit the oral ecosystem nor contribute to the biofilm’s complexity in the same manner. *S. oralis* acts as an initial colonizer within the biofilm, facilitating the adhesion and proliferation of subsequent bacterial populations by modifying the local environment [16]. Due to its vast co-aggregation capabilities, *F. nucleatum*, a secondary colonizer, bridges early and late colonizers [16]. The late colonizer, *A. actinomycetemcomitans*, is closely tied to the pathogenesis of peri-implantitis, contributing to significant inflammatory responses and disease progression [17]. A model encompassing relevant bacteria is indispensable for accurately simulating the dynamic microbial interplay and pathogenic mechanisms underlying peri-implantitis [18]. This approach is particularly pertinent given that peri-implantitis remains a leading complication in dental implantology, with bacterial infections causing bone loss and potential implant failure [19]. Thus, studying these particular bacteria within a multi-species biofilm framework is necessary to advance our knowledge of peri-implantitis and enhance the efficacy of debridement and treatment strategies, ensuring they are tailored to combat the specific microbial challenges presented by this complex condition [20, 21].

The treatment of peri-implantitis consists of surgical or non-surgical sub-marginal instrumentation of the implant, aiming to eliminate the biofilm [22, 23]. However, due to the microscopic and macroscopic characteristics of dental implants, the effects of the treatment of peri-implantitis are not predictable [22]. Some authors hesitate to use certain mechanical treatment options as they may damage the implant surface [24]. As an adjunct to mechanical treatment, chemical decontamination strategies have shown varying results regarding clinical outcomes [25]. Clinicians have little agreement about the most effective treatment methods [26, 27]. Chemical treatments are considered valuable complementary cleansing methods that can be used not only during the established or late stage of the disease but also as preventative measures. However, there is insufficient knowledge about their efficacy [28]. Therefore, evaluating existing methods and developing new treatment methods specially developed for implant surfaces are necessary [29].

Evaluating the impact of various treatment methods on the formation of the pellicle on implant surfaces, which is crucial for biofilm development, can provide valuable insights into strategies to hinder biofilm formation. In particular, investigating the re-formation of the pellicle on the implant surface after decontamination could offer insight into preventing biofilm formation on these surfaces. This study examined several chemical decontamination solutions, assessing their effectiveness in decontaminating the implant surface. These assessments were conducted using a pellicle and multi-species anaerobic biofilm models. The study’s objective was to investigate the impact of six distinct chemical debridement solutions during two early stages critical to developing peri-implant diseases. This included evaluating the efficacy of a novel polymer material in decontaminating the surface. The first stages involved removing the acquired pellicle and assessing the cleaning agent’s effectiveness in preventing pellicle re-formation. The second stage examined the antibacterial effect of an early-matured biofilm using a multi-species subgingival biofilm model.

## Materials and methods

### Preparation of titanium discs and decontamination groups

Commercially, pure titanium discs with a diameter of 6.2 mm and a height of 2 mm were prepared to resemble a rough dental implant surface, mimicking the commercial OsseoSpeed^®^ surface (Dentsply Sirona, Zürich, CH) according to Lamolle et al. [30]. In this protocol, the discs undergo acid etching and are subsequently stored in ethanol prior to use. All surfaces were analysed with a light laser profilometer (PLμ NEOX, Sensofar-Tech S.L., Terrassa, Spain) to ensure a homogenous surface according to previously described procedures [31, 32]. Six decontamination groups were used in both parts of this study (Table 1): ‘EDTA (Prefgel^®^, Straumann AG, Basel, Switzerland), NaOCl (Perisolv^®^, Regedent AG, Zurich, Switzerland), 3% H_2_O_2_ (Sigma-Aldrich, Norway), Poloxamer gel (Pluronic^®^ F-127, Sigma-Aldrich, Norway), Poloxamer gel (Pluronic F-127) + 3% H_2_O_2_ (Nubone^®^ Clean, Corticalis AS, Oslo, Norway) and Chlorhexidine (GUM^®^ Paroex^®^ Sunstar Suisse, Etoy, Switzerland)’.

**Table 1.** Decontamination products used in the study

Nomenclature	Product name	Content	Commercially available
EDTA	PrefGel^®^	24% EDTA + hydrogel	For periodontal use
NaOCl	Perisolv^®^	Sodium hypochlorite (buffered with amino acids) + hydrogel	For implant surface cleaning
H_2_O_2_	Hydrogenperoxide	3% H_2_O_2_ in water	Generic compound
Poloxamer gel	Pluronic^®^ F-127, Sigma-Aldrich	28% Poloxamer in water	For wound-care
Poloxamer + H_2_O_2_	NuBone Clean^®^	3% H_2_O_2_ + hydrogel (Poloxamer, Pluronic^®^ F-127)	In clinical testing for peri-implantitis
CHX	GUM^®^ Paroex^®^	0.12% Chlorhexidine digluconate + 0.05% cetylpyridinium chloride	As mouth rinse

### Dental pellicle model

#### Pellicle formation and decontamination

**Figure 1.**
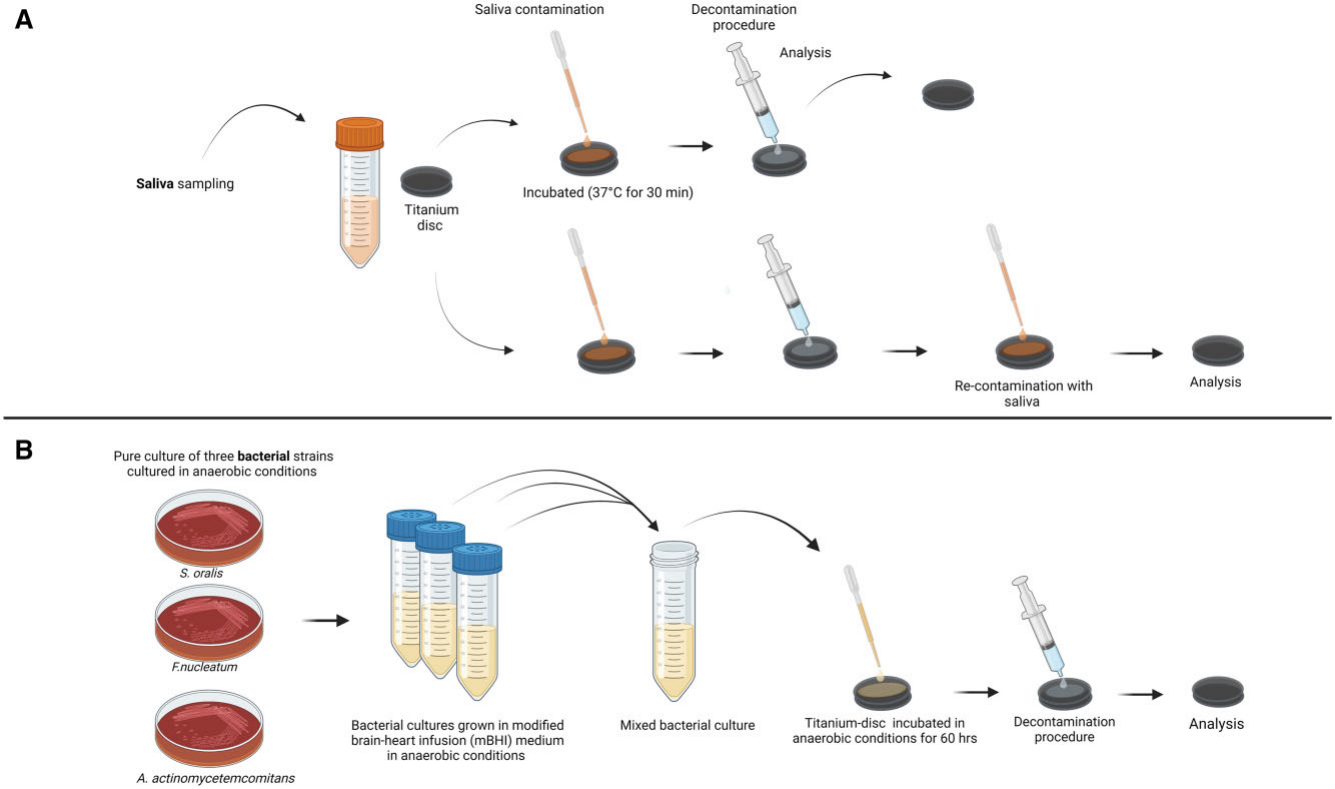
Methodology of the research design. (**A**) Pellicle formation on titanium discs, followed by the appliance of decontamination solutions/gels and a parallel process involving re-contamination of the discs to evaluate the re-formation of pellicle. (**B**) Illustration of the development of a multi-species biofilm model cultivated anaerobically on titanium discs (Figure is made with biorender).

Saliva was sampled from three healthy individuals, pooled, and centrifuged (4000×*g* for 4 min at 20°C) to remove cellular debris and decrease the turbidity, supernatant was used. The prepared titanium discs were placed in 24-well plates (Thermo Fisher, Waltham, USA). For each titanium disc, 2 ml of the pooled saliva was applied to the surface, covering the prepared surface of the titanium disc and incubated at 37°C for 30 min to acquire pellicle formation on the surface. The pellicle was not sterilized. However, bacteria in the pellicle were not assessed. Three discs for each decontamination group were treated with saliva and incubated at three individual times (*n* = 9) to allow pellicle formation. For each essay, two parallel sets of discs were used. One parallel was decontaminated and analysed; the other parallel was decontaminated and then re-contaminated with the pooled saliva and analysed after re-contamination. Figure 1A illustrates that two parallel sets of discs are contaminated to allow pellicle formation, followed by the appliance of decontamination solutions/gels, while the second parallel process involves re-contamination of the discs to evaluate the re-formation of the pellicle after decontamination.

Two millilitres of the decontamination gels/liquids, enough to cover the titanium disc surface, were applied for 2 min and then rinsed for 30 s with ultrapure water (VWR, Oslo, Norway) before analysing the surface. Discs with pellicles (no treatment) were used as a negative control, and discs without pellicles were used as a positive control.

#### Contact angle calculation—analysis of surface hydrophobicity/hydrophilicity

Static contact angle was used to evaluate the hydrophobicity/hydrophilicity of the surface, with the sessile drop method (OCA 20, DataPhysics Instruments GmbH, Filderstadt, Germany) according to the Young–Laplace fitting at room temperature using ultrapure water (VWR, Oslo, Norway) as wetting agent (*n* = 3).

#### Energy-dispersive X-ray spectroscopy and scanning electron microscope

The surface morphology was examined by scanning electron microscopy (SEM) (TM3030, Hitachi, Germany) coupled with electron diffraction analysis [energy-dispersive X-ray spectroscopy (EDX)] with back-scattering electrons at 15 kV. The EDX spectra acquisition time was 150 s; 12-mm double-sided adhesive carbon tabs from Agar Scientific were used to mount the sherd samples on the SEM stage at a 480 µm × 360 µm image size at 2500 magnitude and WD 8.8 mm. The elements titanium, carbon, oxygen and nitrogen were recorded. The EDX were processed with Quantax 70 software (Hitachi, Germany).

#### X-ray photoelectron spectroscopy analysis of chemical surface composition

The X-ray photoelectron spectroscopy (XPS) analysis was conducted on an Axis UltraDLD XP spectrometer (Kratos Analytical Limited, Manchester, UK). The emission of the photoelectrons from the sample was 90° (normal to the sample surface), and the incidence angle of the X-rays was 33.3° (or 56.7° between the X-ray incidence direction and captured photoelectron emission direction). A hybrid lens mode was used with a slot aperture (analysis area of 700 × 300 µm^2^). Survey spectra were acquired with 80 eV pass energy between 0 and 1100 eV binding energy (BE), and detail spectra were recorded for O 1 s, C 1 s, Ti 2p and N 1 s with 40 eV pass energy. The instrument resolution was 1.1 eV for the survey scans and 0.71 eV for the detail scans for the employed settings, determined by measuring the full width at half maximum FWHM of the Ag 3d5/2 peak obtained on sputter-cleaned silver foil. The energy shift due to surface charging was below 1 eV based on the C 1 s peak position relative to the established BEs; therefore the experiment was performed without charge compensation. All samples were referenced to C 1 s at 284.5 eV. The XPS data analysis was performed using the CasaXPS (computer-aided surface analysis for XPS) software package (Casa Software Ltd, Teignmouth, UK).

### Multi-species biofilm model

#### Bacterial strains, biofilm development on the titanium surface and cleaning procedure

A multiple-species biofilm model was used in this study. Strains of an initial colonizer *S. oralis* 11427 NCTC, one secondary colonizer *F. nucleatum* ATCC 10953 and a late colonizer *A. actinomycetemcomitans* DSMZ 8324 were selected. Bacteria were inoculated from frozen stocks onto blood agar (Blood agar Base No. 2 (Oxoid)), supplemented with sterile sheep blood, haemin (0. 5 g/ml) and menadione, and grown under anaerobic conditions (10% H_2_, 10% CO_2_ and balance N_2_) at 37°C for 24–96 h.

For experiments, colonies from blood agar were inoculated into a modified brain heart infusion medium: brain heart infusion (BHI) (VWR, BDH chemicals) supplemented with 2.5 g/l mucin (Merck), 1.0 g/l yeast extract (Oxoid), 0.1 g/l cysteine (Sigma), 2.0 g/l sodium bicarbonate (Merck), 5.0 mg/ml haemin (Sigma), 0.1 mg/L menadione (Merck) 1.0 mg/ml and 0.1 g/l glutamic acid (Sigma) [33].

Growth of pure cultures of each species in the modified BHI medium under anaerobic conditions was analysed using optical density measurements and colony-forming units (CFU) counting. Bacteria were collected in mid-exponential growth, and the bacterial suspensions were diluted in fresh modified BHI (*S. oralis* to 10^3^ CFU ml^−1^, *F. nucleatum* and *A. actinomycetemcomitans* to 10^6^ CFU ml^−1^). A mixed bacterial suspension was prepared by combining equal volumes of the three individual suspensions.

1.5 ml of the mixed bacterial suspension was applied to the prepared titanium discs in a sterilized 24-well culture plate (Thermo Fisher), and the plates were incubated under anaerobic conditions (10% H_2_, 10% CO_2_ and balance N_2_) at 37°C for 60 h (Figure 1). Wells containing culture medium only were included to control for sterility of the medium. After incubation, the discs (*n* = 9) were carefully removed from suspension, rinsed with 2 ml of sterile phosphate buffer saline (PBS) to remove non-adherent bacteria and placed in a new 24-well plate. The decontamination solutions were applied for 2 min and then rinsed in 2 ml of sterile PBS before analyses.

All experiments were conducted in an anaerobic chamber, Witley A35 workstation (Don Whitley Scientific, West Yorkshire, UK).

#### Analysis of biomass of the biofilm

The biofilm biomass was determined by staining the adherent bacteria on the discs with 0.1% safranin (Merck) for 30 min. The unabsorbed safranin and unattached bacteria were removed by washing in PBS. Safranin was released from the biofilm by incubation in acetic acid (30%) for 10 min, and the amount of safranin was quantified by measuring the absorbance at 530 nm in a Cytation™ 3 Cell Imaging Multi-Mode Reader (BioTek, Santa Clara, CA, USA). The experiments were repeated three times.

Discs incubated in sterile modified BHI were used as a positive control, and discs with biofilm (no decontamination) were used as a negative control.

#### SEM—morphological analysis of biofilms

The morphological analysis of biofilms was performed using SEM. Specimen fixation involved double-strength fixation with PHEM (Pipes-Hepes-EGTA-Magnesium) buffer (2xPHEM, 1% glutaraldehyde, and 4% paraformaldehyde) for 15 minutes at the growth temperature, followed by storage at 4°C until use. The discs were critical point dried, sputter-coated with gold, and analysed using an S-4800 SEM by applying systematic uniform randomized sampling at an image resolution of 5 kV. Fifteen SEM images were acquired for each sample. Additionally, detailed high-magnification images of areas of interest were taken. Three discs per group were evaluated, and discs with biofilm without decontamination were used as a negative control.

#### Confocal laser scanning microscopy—analysis of biofilm vitality and thickness

Biofilm images were collected by confocal laser scanning microscopy (CLSM) using a LSM 510 confocal scanning system (Zeiss, Carl Zeiss Jena, Germany). Treated discs were dipped in 0.9% NaCl to eliminate weakly attached cells, and the biofilms were stained with the Live/Dead Bac Light Bacterial Viability kit for microscopy (L7007, Molecular Probes, Invitrogen). At each disc, at least three different and representative locations were selected. A z-series of scans (xyz) were analysed using Zen software to measure the z-thickness (in µm). Biomass and cell viability within the biofilm were quantified using ImageJ, using the manual counting tool, where voxel intensities from two channels were measured, and cell viability within the stacks was calculated. Discs with biofilm and without treatment were used as a negative control.

### Statistical analysis

A normality test was performed prior to analysis, and only a limited number of variables exhibited normal distribution; hence, non-parametric analysis was conducted. Data are summarized and expressed as medians and interquartile ranges (IQR). The significance level was evaluated using non-parametric Kruskal–Wallis ANOVA. In addition, the Mann–Whitney *U* test was used to control the results between the tested disinfection procedure and the control. Prism 8 (GraphPad Software, San Diego, CA, USA) and StataSE 17 (StataCorp, College Station, Texas, USA) were used for statistical analysis. A *P* value < 0.05 was considered statistically significant in all analyses.

## Results

### Elemental analysis of titanium after contamination and decontamination using EDX imaging

**Figure 2.**
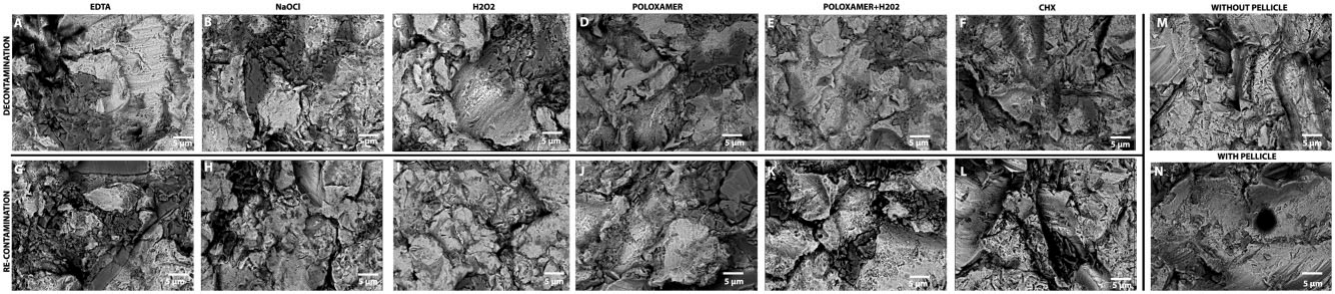
SEM images of titanium implant surfaces following pellicle contamination and respective decontamination treatments with various strategies. The top panels (**A**–**F**) illustrate the initial contamination phase, while the bottom panels (**G**–**L**) display the re-contamination phase. Right panels are controls. Scale bars represent 5 µm.

The SEM micrographs present titanium implants’ detailed surface morphology after contamination with a proteinaceous pellicle and subsequent re-contamination phases (Figure 2). The Top row (Figure 2A–F) shows the titanium surface after removing the pellicle with various chemical debridements and the lower panels after pellicle re-recontamination (Figure 2G–L). Two controls are: without pellicle (Figure 2M) and with pellicle (Figure 2N). The micrograph-labelled ‘H_2_O_2_’ (Figure 2C for decontamination, Figure 2I for re-contamination) displays a rugged topography with varying degrees of surface coverage. The very dark areas, presumed to be organic residues, are scattered across the surface, indicating incomplete removal of the pellicle. The decontaminated surface (Figure 2C) reveals patches where the underlying titanium appears to be exposed, yet interspersed with regions where the dark contrast suggests the persistence of organic material. However, it is difficult to distinguish between the group from the SEM images. The re-contaminated surface (Figure 2I) shows an increase in the dark areas, which implies that the surface has undergone additional contamination, possibly due to the re-adhesion of proteins or other organic compounds. In contrast, the panels representing the ‘Poloxamer + H_2_O_2_’ treatment (Figure 2E for decontamination, Figure 2K for re-contamination) exhibit a notably different surface morphology. The decontaminated surface (Figure 2E) shows a reduced presence of very dark areas, suggesting a more effective removal of the organic pellicle. The surface seems more uniform, with fewer and smaller patches of dark contrast, indicative of a cleaner titanium substrate. Upon re-contamination (Figure 2K), there appears to be a slight resurgence of dark regions, yet they are markedly less pronounced compared to the ‘H_2_O_2_’-only treatment, indicating a lower level of re-contamination.

**Figure 3.**
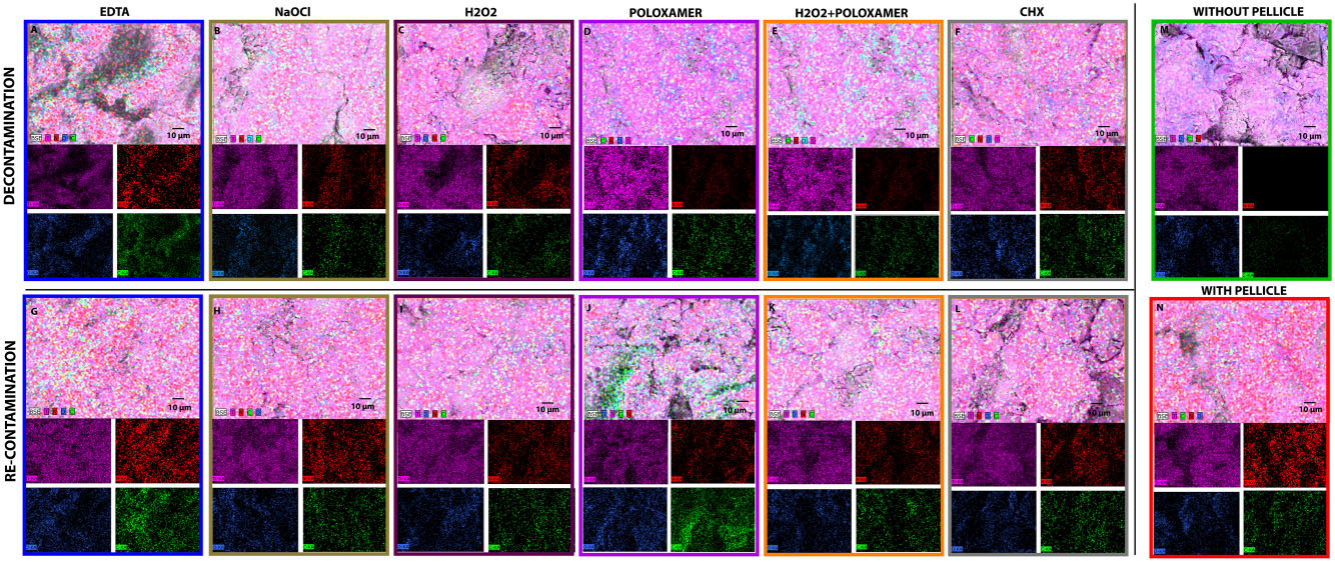
Comparative EDX elemental mapping of titanium implant surfaces after contamination and subsequent decontamination treatments. The top row (**A**–**F**) represents the initial decontamination phase, while the bottom row (**G**–**L**) illustrates the re-contamination phase. Positive and negative controls to the right. The elemental composition is colour-coded with titanium (Ti) in magenta, indicating the extent of surface cleanliness; oxygen (O) in blue, nitrogen (N) in green, signifying the presence of protein contaminants; and carbon (C) in red, representing organic contamination or residual cleaning substances. The scale bar in the images corresponds to 10 µm.

Upon examination of the EDX images, a qualitative assessment of elemental distribution on titanium implant surfaces after decontamination and re-contamination with pellicle is presented. In these images, the presence of titanium, indicated by the magenta colouration, serves as a proxy for the cleanliness of the surface (Figure 3). The top row (Figure 3A–F) shows the titanium surface after removing the pellicle with various chemical debridements and the lower panels shows surface after re-contamination with pellicle (Figure 3G–L). Two controls are: without pellicle (Figure 3M) and with pellicle (Figure 3N). A surface with a uniform and intense magenta hue denotes a predominantly titanium presence, indicating minimal contamination. Conversely, deviations from this magenta dominance suggest the adherence of extraneous substances. Nitrogen, visualized in green, denotes the presence of proteins, which are considered contaminants in the context of implant surfaces.

The prevalence of green areas within the images correlates with higher concentrations of proteinaceous material, an unwanted outcome following decontamination procedures. The carbon signal, represented in red, is more ambiguous due to its association with both protein contaminants and the possible remnants of hydrogel used in cleaning the titanium surfaces. Consequently, interpreting carbon presence requires careful consideration of the cleaning agents used and the expected background levels of carbon-based contaminants. The highest level of red was seen for the ‘EDTA’ group (Figure 3A and G) and also increased for ‘NaOCl’ (Figure 3 and H). The nitrogen levels were highest for the re-contamination procedure, and poloxamer (Figure 3G) and ‘EDTA’ (Figure 3J) had the most profound green colour. The images corresponding to the ‘H_2_O_2_’ treatment (Figure 3C and I) show a modest retention of magenta, indicating that while some areas of the titanium surface are clear of organic contaminants, a significant fraction remains covered. The nitrogen signal is prevalent, suggesting a substantial presence of proteinaceous material, which is an undesired outcome post-decontamination. The carbon distribution appears moderately intense, suggesting the presence of organic contaminants or residual cleaning agents. The EDX images for the combined ‘H_2_O_2_’ and ‘Poloxamer’ treatment (Figure 3E and K) reveal a more pronounced magenta hue, suggesting a cleaner titanium surface with reduced contamination. While still present, the green nitrogen signal is less intense than that of the ‘H_2_O_2_’-only group, indicating a more effective reduction of protein contaminants. The red carbon signal is also diminished, which may reflect a more thorough cleaning effect, reducing both protein contaminants and residual hydrogel substances. The comparative analysis between the ‘H_2_O_2_’ and ‘Poloxamer + H_2_O_2_’ treatments elucidates the enhanced efficacy of the combined agents in restoring the titanium surface. The improved cleanliness is visually evidenced by the increased magenta saturation and decreased green and red intensities in the ‘Poloxamer + H_2_O_2_’ group. This suggests that the synergistic effect of the combined cleaning agents more effectively eliminates protein-based contamination and residual organic material.

### Analysis of surface hydrophobicity/hydrophilicity

**Figure 4.**
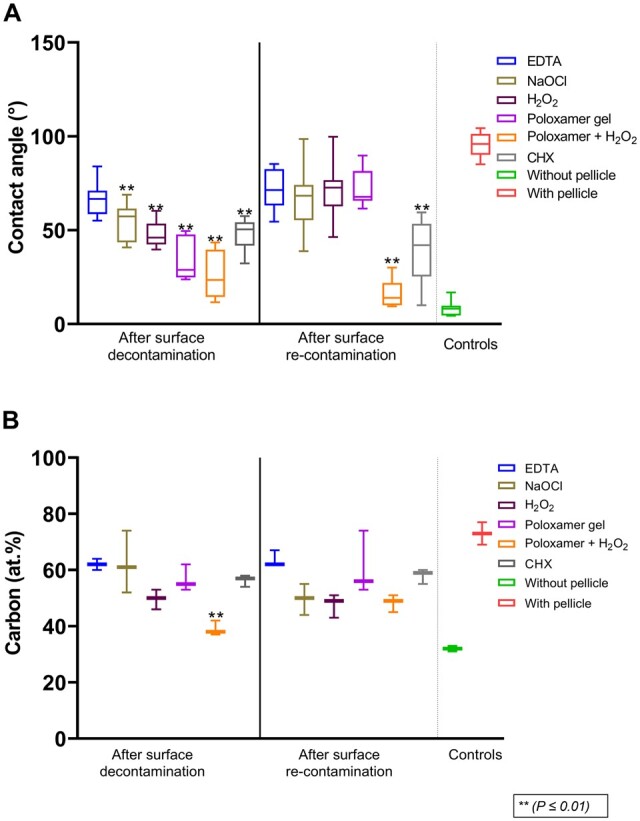
(**A**) Contact angle measurements and (**B**) carbon concentration (atomic %). ** marks a significant difference from negative control.

Treatment with all of the tested solutions (‘NaOCl’: 57.4 (IQR 43.6–61.5), ‘H_2_O_2_’: 46.0 (IQR 42.3–53.5), ‘poloxamer’: 28.9° (IQR 24.8–47.7), ‘poloxamer + H_2_O_2_’: 23.5° (IQR 14.5–39.6), ‘CHX’: 50.5° (IQR 41.7–54.3)) except ‘EDTA’ (66.6° (IQR 58.6–71.0), *P* = 0.53) resulted in a statistically significant reduction (*P* ≤ 0.01) in contact angle compared to the untreated control (i.e. surface with pellicle: 95.9° (IQR 90.1–101.3)), indicating pellicle reduction. In the pellicle re-contamination phase, only ‘poloxamer + H_2_O_2_’ (14.0° (IQR 10.0–21.9), *P* ≤ 0.01) and ‘CHX’ (42.0°, (IQR 25.4–53.4) *P* ≤ 0.01) exhibited a significantly lower contact angle compared to the untreated control (Figure 4A). ‘Poloxamer + H_2_O_2_’ provided the most hydrophilic surface, both after decontamination (23.5° (IQR 14.5–39.6), *P* ≤ 0.01) and after pellicle re-contamination (14.0° (IQR 10.0–21.9), *P* ≤ 0.01; Figure 4A).

### Analysis of chemical surface composition by XPS

**Figure 5.**
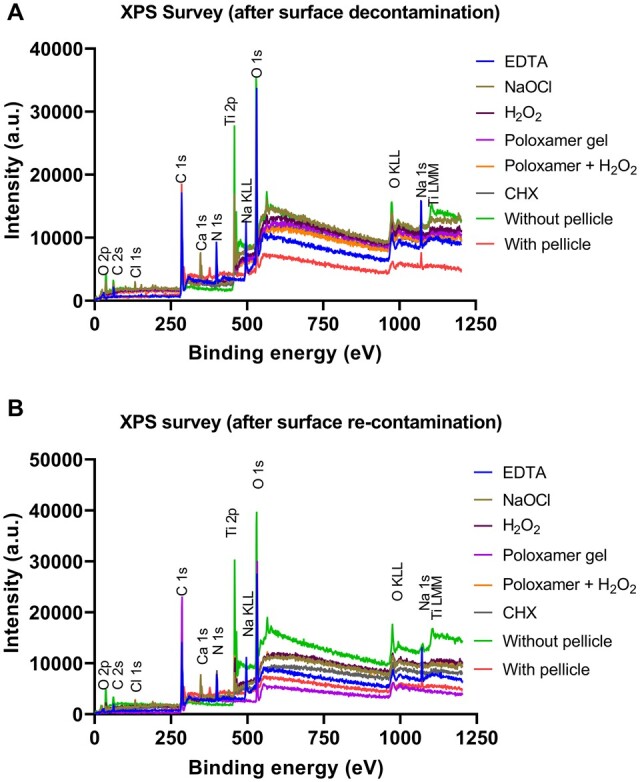
(**A**) XPS Survey spectra between 0 and 1200 eV binding energy after surface decontamination. (**B**) XPS survey spectra between 0 and 1200 eV binding energy after surface re-contamination.

XPS provided a detailed surface chemistry analysis on atomic level measurement after pellicle removal and re-establishment of pellicle (Figure 5A and B). ‘Poloxamer + H_2_O_2_’ (38% (IQR 37–42)) demonstrated a statistically significant difference (*P* ≤ 0.01) in the atomic percent (%) concentration of carbon from the untreated control (73% (IQR 69–77)) after pellicle decontamination*.* None of the other groups showed a significant difference from the untreated control, (Figure 4B)*.* It was lower for ‘NaOCl’ (50% (IQR 44–55)), ‘H_2_O_2_’ (49% (IQR 43–51)) and ‘Poloxamer + H_2_O_2_’ (49% (IQR 45–51)) compared to the other groups (EDTA: 62% (IQR 62–67), ‘Poloxamer’: 56.0% (IQR 53–74), CHX: 59.0% (IQR 55–60)) in the re-contamination phase; however, this effect was not statistically significant different from the untreated control as shown in Figure 5B.

### Analysis of biomass of the biofilm

**Figure 6.**
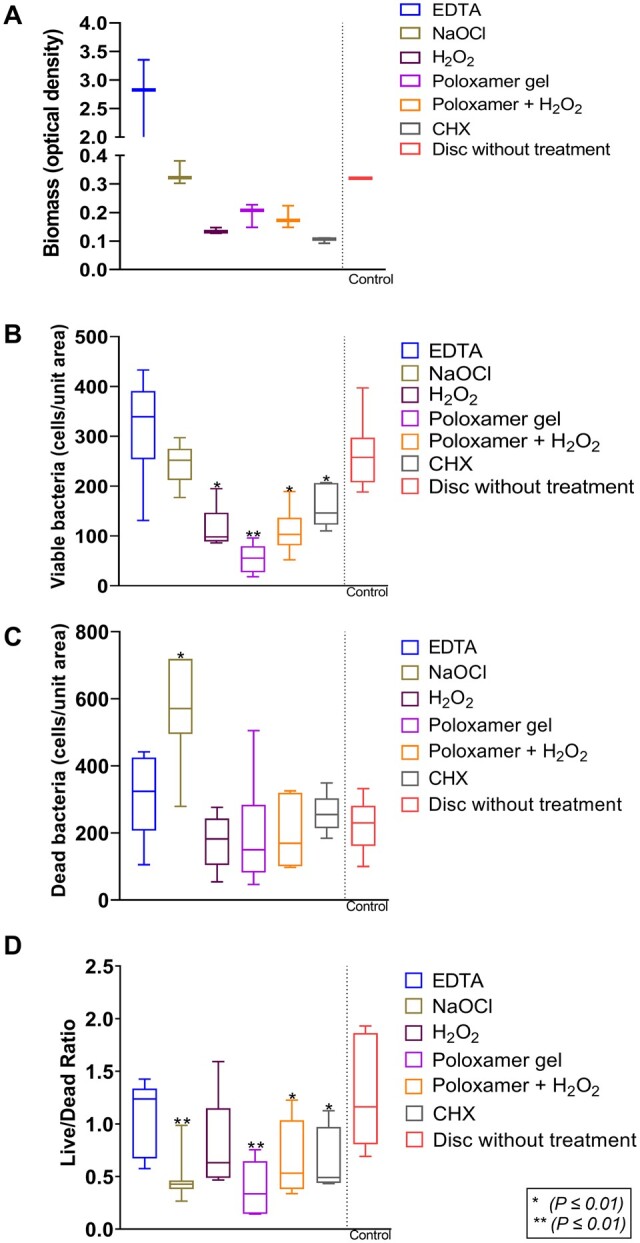
(**A**) Biomass of biofilm, (**B**) count of viable bacteria cells, (**C**) count of dead bacteria cells and (**D**) live/dead ratio. * and ** mark a significant difference from the negative control.

The quantitative analysis of the amount of biofilm biomass using safranin staining was compared between the groups (‘EDTA’: 2.83 (IQR 0.74–3.35), *‘*NaOCl’: 0.32 (IQR 0.30–0.38), ‘H_2_O_2_’: 0.13 (IQR 0.13–0.15), ‘Poloxamer’: 0.21 (IQR 0.15–0.23), ‘Poloxamer + H_2_O_2_’: 0.17 (IQR 0.15–0.22), CHX: 0.11 (IQR 0.09–0.11)). Figure 6A shows a tendency of lower biofilm mass compared to the opposing group for all groups except for ‘NaOCl’ and ‘EDTA’. However, no statistical significance was shown (Figure 6A).

### CLSM—analysis of biofilm vitality and thickness

Viable biomass (cells/unit area) compared to untreated biofilm was significantly lower in the following groups: ‘H_2_O_2_’ (98 (IQR 89–146), *P* ≤ 0.05), ‘Poloxamer’ (55 (IQR 27–80), *P* ≤ 0.01), ‘Poloxamer + H_2_O_2_’ (103 (IQR 81–137), *P* ≤ 0.05) and ‘CHX’ (146 (IQR 123–206), *P* ≤ 0.05). Biofilms treated with ‘EDTA’ (339 (IQR 254–391)) or ‘NaOCl’ (252 (IQR 212–275)) did not have a significantly lower number of viable cells compared to the untreated control (257 (IQR 207–297); Figure 6B).

‘NaOCl’ (571 (IQR 495–719)) showed a significantly higher number of dead bacteria (cells/unit area) compared to the untreated control group (230 (161–281)), *P* ≤ 0.05 (Figure 6C). None of the other groups showed any statistically significant difference compared to an untreated surface. The Live/Dead ratio median was under 1 for all remedies except for ‘EDTA’ (Figure 6D).

**Figure 7.**
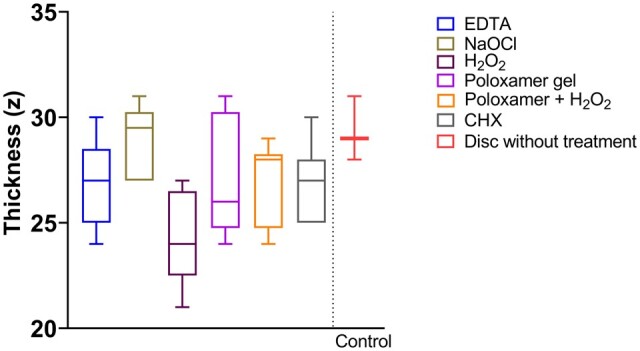
Thickness (*z*) of biofilm.

The thickness of the biofilm ranged between 20 and 31 µm (Figure 7), with no statistically significant difference between treatment categories compared to the untreated control group (i.e. disc with biofilm).

### Morphological analysis of biofilms

**Figure 8.**
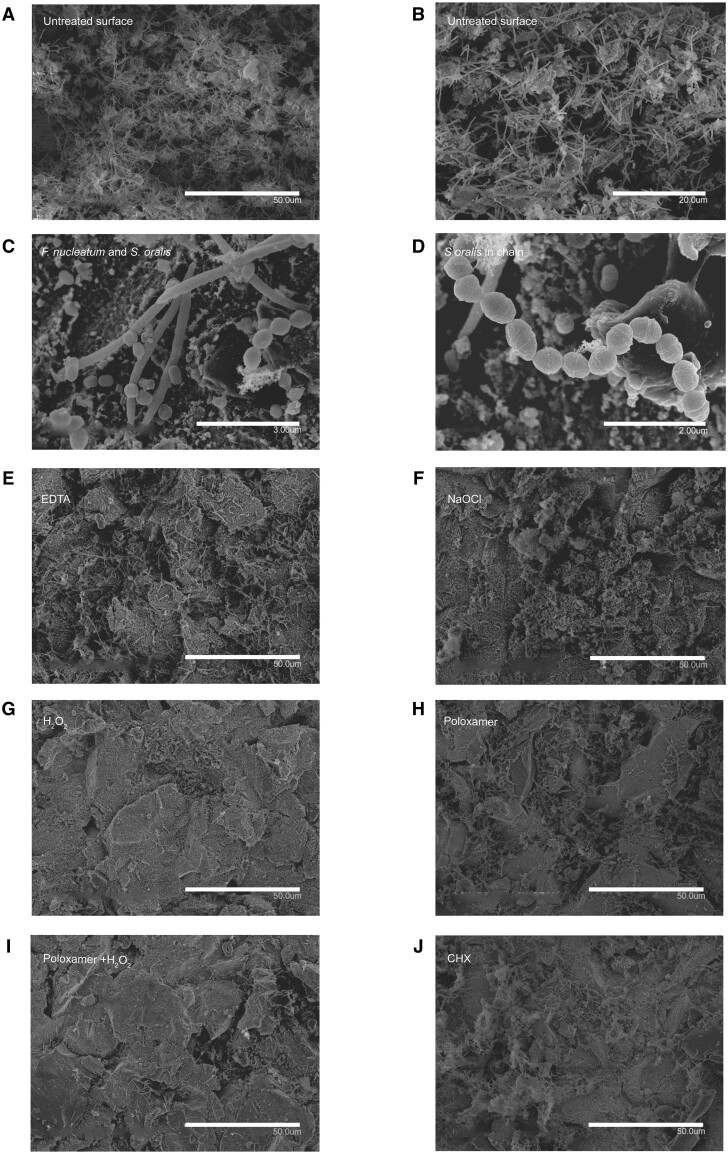
Scanning electron microscopy images of biofilms after chemical decontamination. Untreated surface (**A** and **B**) and bacterial stacks and tunnel formation are seen (**F**). Nucleatum can be easily located in the image due to its slender cells and tapered end morphology (**C**), while *S. oralis* are arranged in chains (**D**). Surface treated with EDTA (**E**), NaOCl (F), H_2_O_2_ (**G**), Poloxamer (**H**), Poloxamer + H_2_O_2_ (**I**) and CHX (**J**). Magnification: (A, E, F, G, H, I, J) 1000×, (B) 2000×, (C) 15 000× and (D) 22 000×.

The negative control group showed a typical mature biofilm with evident bacterial stacks and tunnel formation (Figure 8A and B). The three strains used in the experiment were also identified within the dense biofilm, with *S. oralis* and *F. nucleatum* (Figure 8C and D) being more accessible to locate than *A. actinomycetemcomitans*, which grows in aggregates. Fifteen standardized uniformed randomization (SUR) images were taken for each group, and while none of the groups were utterly free from biofilm, morphological analysis revealed some differences between the groups. The discs treated with ‘EDTA’ exhibited a significant amount of biofilm, but some areas without biofilm were also observed (Figure 8E). With ‘NaOCl’ treatment, biofilm disruption was observed in some areas, but connected and stacked biofilm was seen in other regions (Figure 8F). The biofilm appeared visually more densely packed. ‘H_2_O_2_’ treatment resulted in more disrupted surfaces and many areas without biofilm. However, bacteria were present in the microstructures and attached to the surface (Figure 8G). The ‘Poloxamer + H_2_O_2_’ group showed a similar morphology to the ‘H_2_O_2_’ treatment (Figure 8I). In the case of ‘Poloxamer’, some areas with disrupted biofilm were observed, but many areas with present biofilm were also seen (Figure 8H). Figure 8J displays ‘CHX’ treatment; this resulted in numerous areas with biofilm, which appeared more compact than in the other groups.

## Discussion

Bacteria are prone to attach to biomaterial surfaces and may form biofilm. These biofilms are complex communities of microorganisms, primarily bacteria, which adhere to biomaterial surfaces and are embedded within a self-produced matrix of extracellular polymeric substances [34–36]. The type of bacteria and biofilm varies from biomaterial to biomaterial and depends on many factors, such as type of material, surface roughness and chemistry, and implantation location. Biofilms that can form on bone implants, leading to infections that are difficult to treat, are anaerobic and contain several species. Literature on antibacterial dental implants typically uses strains like *S. epidermidis* [37–42] or *Staphylococcus aureus* [43–49]. However, these bacteria types are absent in peri-implantitis and, therefore, are not clinically relevant when examining the antibacterial effect [17, 50, 51]. Although the latter are common in hospital-acquired infections, they are not typical inhabitants of the oral cavity and do not contribute significantly to the complexity of oral biofilms [17]. It is essential to utilize a multi-species biofilm model incorporating key oral bacteria like *S. oralis*, *F. nucleatum* and *A. actinomycetemcomitans* [13–15] to gain a comprehensive understanding of peri-implantitis. These bacterial species and their involvement make them more relevant for studies of peri-implantitis than other bacteria. The anaerobic environmental conditions employed in this study’s biofilm model are also significant, reflecting the anaerobic environment present in inflamed pockets around implants. The significance of multi-species biofilms in various applications is well recognized, yet understanding interspecies dynamics within these biofilms remains substantially underexplored. It is unclear whether these interactions are characterized by competition, mutual cooperation or a balance of both. However, existing evidence underscores the imperative for a more in-depth investigation of these interspecies interactions, emphasizing a holistic approach to the biofilm community rather than isolating its individual constituents [52]. Therefore, it is imperative to use clinically relevant multi-species biofilm models when investigating the antibacterial effect of dental biomaterials and ensure effective translation into the clinic.

These infections are a significant concern in dental implant surgery because they can lead to implant failure, necessitating implant removal and replacement, which is costly and burdensome for the patient. In biomaterial science, the challenge is to develop implant surfaces and coatings that resist biofilm formation while promoting healthy integration with the surrounding bone tissue [53–56]. This includes researching bacteriostatic or bactericidal materials, developing surface topographies discouraging bacterial adhesion, and incorporating drug-eluting properties to prevent or treat biofilm-related infections [57–64]. These innovations are crucial for the long-term success of bone implants and the overall health and recovery of patients. However, studies have shown that different dental implant surfaces do not change the outcome of peri-implantitis, and the drug-eluting properties are usually gone when the peri-implantitis disease starts to develop (typically after 5 years) [65–68]. Therefore, the use of debridement techniques is essential.

Multiple chemical remedies have been used in dentistry for a long time, and many of these remedies are now adopted and used for cleaning an implant surface. Our study compares these remedies to the effect on biofilm removal and pellicle formation on an implant surface. The efficacy of available treatments on an implant surface is insufficient, as evidenced by the findings of this current laboratory study. Consequently, developing novel remedies is highly important and is expected by experts in the field [26, 29]. The best overall results in this study appear to come from the ‘Poloxamer gel’ activated with ‘H_2_O_2_’. ‘Poloxamer’ is a class of triblock copolymers consisting of poly(ethylene oxide) (PEO)- and poly(propylene oxide) (PPO)-blocks, which have the general structure PEO–PPO–PEO. It has been utilized as a wound cleanser for chronic wounds with delayed healing, resulting in positive outcomes [69, 70]. When activated with hydrogen peroxide, the organic molecules become surfactants that rapidly form micelles, making the poloxamer act as a strong, non-ionic detergent, helping to emulsify and solubilize organic contaminants [70, 71], thereby facilitating their removal from the titanium surface as observed by a decrease in carbon contaminates as seen in the XPS analysis. XPS is crucial in analysing proteins adsorbed on titanium surfaces, offering advantages over EDX. XPS’s surface sensitivity, with an analysis depth of around 10 nm, is ideal for examining thin protein films on titanium [72, 73]. Unlike EDX, which provides a basic elemental composition, XPS offers detailed insights into elements’ chemical states and environments, which is essential for understanding protein–titanium interaction mechanisms [74]. Furthermore, XPS can identify changes in binding energies, revealing interactions such as adsorption or bond formation at the interface [75]. Therefore, while EDX is useful for elemental analysis, XPS is indispensable for a comprehensive understanding of protein–titanium interactions.

In accordance with the XPS analysis, SEM observations suggest that the combined use of ‘Poloxamer + H_2_O_2_’ is more effective in decontaminating the titanium surface and potentially provides a more resistive barrier against re-contamination than ‘H_2_O_2_’ alone. The darker areas, indicative of organic residues, are substantially reduced in the ‘Poloxamer + H_2_O_2_’-treated surfaces. This finding is significant for developing effective cleaning protocols for titanium implants, where reducing organic residue is critical for implant success. The SEM images corroborate the notion that a synergistic approach to decontamination, employing both oxidative and surfactant mechanisms, can enhance the cleanliness of the implant surface and may improve the biocompatibility and longevity of the implant in a clinical setting. This result was confirmed by the EDX images, which provided visual evidence supporting the superior performance of the combined ‘Poloxamer + H_2_O_2_’ treatment in decontaminating titanium implant surfaces. This treatment results in a cleaner surface with less protein contamination and residual carbon-based substances, as indicated by the colourimetric mapping of the elemental distribution. These findings highlight the potential benefits of combining decontamination to achieve an optimal titanium surface for clinical applications.

In the oral cavity, the pellicle forms on the implant surface within seconds after cleaning exposed implant components. Bacterial adhesion and biofilm development depend on the composition and adhesion strength of the proteins adhering to the surface [76]. The composition and adhesion strength of the proteins varies and is influenced by several factors, including surface chemistry and charge [77]. Cleaning an exposed implant aims to provide a reactivated surface that promotes tissue regeneration and impedes bacterial colonization.

Hydrophilicity is a property determined by the chemical composition of the surface [78], and all remedies used for treating an implant surface will affect it. The contact angle measures surface hydrophilicity; a clean titanium surface is super-hydrophilic and has a contact angle close to zero [79]. Our findings suggest that all of the gels tested, except for ‘EDTA’, were effective in removing the initial pellicle layer, but only ‘Poloxamer + H_2_O_2_’ and ‘CHX’ prevented its re-formation. It is essential to consider the atomic presence of carbon on the surface in conjunction with the contact angle, as it indicates surface contamination levels [80]. Considering this, ‘Poloxamer + H_2_O_2_’ treatment resulted in the cleanest titanium surface compared to the other chemical debridement agents.

These findings are relevant when considering the treatment of peri-implant diseases. Hydrophilic surfaces attract the necessary cells and proteins for bone formation, particularly in the early stages of bone formation [81–83]. Although natural bone regeneration may not always be possible in cases of peri-implantitis, early detection and prevention of the disease can promote bone gain. Because of the damaging results of peri-implantitis and the uncertainty attached to the treatment, both in light of ceasing development of the disease and recovery after the treatment, it is in the patient’s best interest to treat peri-implantitis and peri-implant mucositis at an early stage [84, 85]. However, the choice of remedy should also be combined with the ability to remove biofilm, as the implant surface must remain free of biofilm.

Residual bacterial colonies and biofilm were present in all groups, even those that showed the best cleaning results. This indicates that chemical cleaning alone is insufficient for completely removing biofilm. Mechanical and chemical cleaning should be performed to ensure complete biofilm removal and prevent disease progression, even in the early stages of disease development [86]. However, some reports suggest that invasive mechanical debridement can alter the implant’s surface topography, potentially affecting its mechanical properties and, hence, its ability to promote bone re-osseointegration [24, 87].

When analysing the biofilm biomass, none of the groups showed a statistically significant difference compared to the negative control group, with most of the values being lower than the control, except for ‘EDTA’, which did not seem to affect biofilm removal. The lack of effect on the biofilm from ‘EDTA’ raises concerns about using treatments developed initially for periodontal treatment in peri-implant treatment, emphasizing the need for remedies specifically tailored to the unique properties of implant surfaces. In the same sense, the viable cell load of the biofilm was lower than the control group for all of the groups except for ‘EDTA’ and ‘NaOCl’, indicating their low bactericidal effect on the biofilm tested.

Although these models provide a valuable reference for comparing different decontamination strategies, their clinical translation can be challenging. First, it is an *in vitro* study with only three bacteria to mimic the subgingival/submucosal biofilm environment. Moreover, residual bacteria in microscopic pits are of uncertain clinical relevance, as completely removing bacteria may not always be necessary to heal peri-implant lesions. In addition, the host response, which is not analysed in this model, may play a relevant role [88].

Our study found that the ‘Poloxamer gel’ combined with ‘H_2_O_2_’ showed synergetic effects on the pellicle, but the impact on the biofilm was similar to that of hydrogen peroxide alone. However, the combined effect on the biofilm and the pellicle may provide a beneficial cleaning outcome. In future studies, it would be valuable to evaluate the proteins that adhere to the implant surface after treatment to determine if proteins that are beneficial for regeneration or biofilm formation are present. In addition, more research is needed to evaluate the clinical effectiveness of these remedies through controlled randomized clinical trials and their combination with mechanical treatment methods.

## Conclusion

This comprehensive study provides critical insights into the efficacy of various chemical decontamination strategies for managing peri-implant diseases, mainly focusing on their impact on the acquired pellicle and multi-species biofilms on titanium implant surfaces. The investigation revealed that while no single agent achieved complete biofilm eradication, combining poloxamer gel with H_2_O_2_ (NuBone^®^ Clean) emerged as a potential strategy for decontaminating implant surfaces, demonstrating a synergistic effect that surpassed the individual components. This combination significantly reduced viable bacterial counts and achieved a lower pellicle re-contamination rate, indicating its potential as an effective decontamination method.

The study emphasized the importance of tailoring treatment methods to the unique challenges posed by the diverse microbial populations in peri-implant diseases. The findings from this research are crucial for guiding clinical practices and future studies, as they offer a foundation for developing more effective and targeted treatments for peri-implant diseases, ultimately improving patient outcomes in dental implant care. The research underscores the necessity of combining both mechanical and chemical approaches for optimal management of peri-implant diseases, recognizing the limitations of chemical treatments alone in completely eradicating complex biofilms.

Future research should refine and enhance these chemical strategies and integrate them effectively with mechanical debridement methods. Additionally, clinical trials are needed to validate the effectiveness of these strategies in real-world scenarios, paving the way for more effective, evidence-based approaches in peri-implant disease management.

## Data Availability

The data supporting this study’s findings are available upon reasonable request.
